# Visualization of endogenous NID-1 and EMB-9 in *C. elegans*

**DOI:** 10.17912/micropub.biology.000110

**Published:** 2019-04-19

**Authors:** Kanae Matsuo, Akihiro Koga, Shinji Ihara

**Affiliations:** 1 Department of Chemical and Biological Engineering, Ariake National College of Technology, 150 Higashihagio-machi, Omuta, Fukuoka 836-8585, Japan.

**Figure 1:**
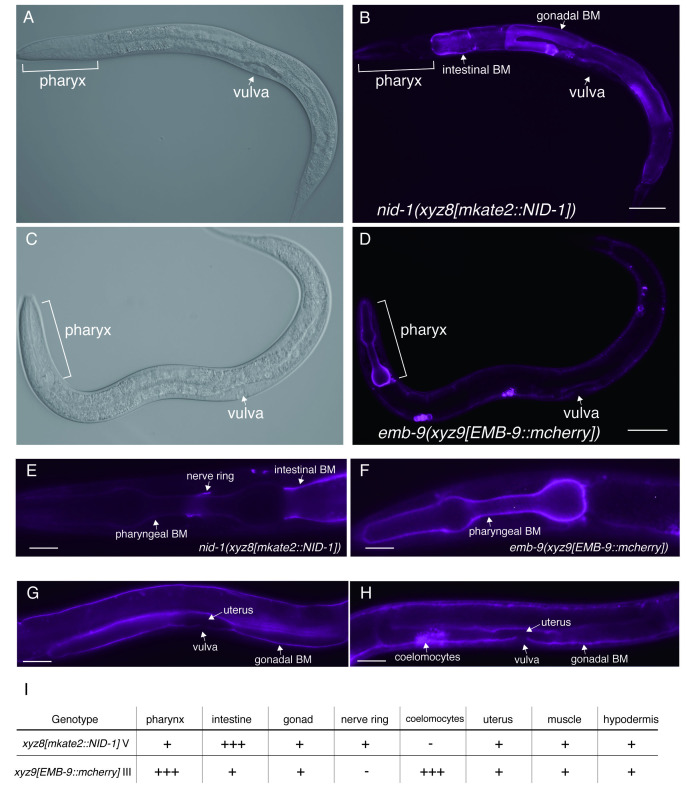
Localization patterns of mkate2::NID-1 and EMB-9::mcherry. (A and B) The localization of mkate2::NID-1 (right), and differential interference contrast (DIC) images (left) of *nid-1(xyz8[mkate2::NID-1])* at L4 stage. (C and D) The localization of EMB-9::mcherry (right), and differential interference contrast (DIC) images (left) of *emb-9(xyz9[emb-9::mcherry])* at L4 stage**.** Scale bars: 50 μm. (E and F) High magnification images of mkate2::NID-1(left), and EMB-9::mcherry(right) in the pharynx at L2 stage. Scale bars: 10 μm. (G and H) High magnification images of mkate2::NID-1(left), and EMB-9::mcherry (right) in the developing vulva of L3 stage. Scale bars: 20 μm. Anterior and dorsal surfaces are to the left and top, respectively. (I) Intensities of mkate2::NID-1 and EMB-9::mcherry at BM in different tissues are indicated; +++, + and – indicate highly detected, detected and faintly or not detected, respectively.

## Description

*nid-1* and *emb-9* are the worm homologs of the human nidogen and human type IV collagen, respectively (Kramer, J. M. 2005). Nidogen is a glycoprotein that can bind type IV collagen with high affinity. *nid-1* is single nidogen gene in the *C. elegans* genome, generating three alternative splice variants (nid-1A, nid-1B, and nid-1C) (Kang & Kramer, 2000). Type IV collagen is the principal component of basement membrane (BM), a complex network of triple helical molecules. *emb-9* encodes sole α1-like type IV collagen that forms a heterotrimer with one α2-like type IV collagen (*let-2*). Nidogen and type IV collagen are components of BM that cover the basal surfaces of nearly all animal tissues. Previous work in *C. elegans* has confirmed the localizations of NID-1 and EMB-9 by using a transgenic strain or immunohistochemistry (Graham *et al.* 1997; Kim & Wadsworth, 2000) . To address endogenous localization patterns of both proteins *in vivo*, we used CRISPR/CAS9 technology to insert fluorescence label at the *nid-1* and *emb-9* genomic locus, and established worms expressing the mkate2::NID-1 or EMB-9::mcherry, respectively. To visualize all isoforms, nid-1A, B, and C, we inserted mkate2 into exon 2, which is a common exon for all isoforms. We found that mkate2::NID-1strongly localized to intestinal BM (Figure 1B and E) and localized to gonadal BM and nerve ring (Figure 1E and G), consistent with previous reports. Interestingly, we found that EMB-9::mcherry strongly localized to BM in pharynx (Figure 1D and F), although mkate2::NID-1 was faintly localized at pharyngeal BM (Figure 1E). We also confirmed strong expression of EMB-9::mcherry in coelomocytes, but not in the strain expressing mkate2::NID-1 (Figure 1G and H). The mkate2::NID-1 and EMB-9::mcherry are similarly localized to BM at gonad and uterus (Figure 1G and 1H). Differences of localization intensity at BM in the tissues are summarized in Figure 1G. Our work indicated that endogenous NID-1 and EMB-9 show different intensities at BM of pharynx, intestine, although it remains unclear whether these differences are associated with organ function.

## Reagents

N2 (Bristol) was used for the injection strain. Animals were cultured on standard NGM plate with *E. coli* (OP50) and maintained at 20 °C. To achieve genome-edited mkate2 knock-in *nid-1* locus, we used pDD287 vector for repair template. pDD287 was modified to generate N-terminal mkate2::self-exiting cassette system repair template using following primers: 5’ *nid-1* fwd AACGACGGCCAGTCGGATAATGTGAGTTTTTATCCAA, 5’ arm *nid-1* rev GGCTCCCGATGCTCCAATTCCATTGTGTGAGAGAAAG, 3’ arm *nid-1* fwd AGCGAGGAAGACTTGGTCGGTCTTGGAAAACCGACGC, and 3’ arm *nid-1* rev CTATGACCATGTTATCTGATTATTGGACAAACTGAAA. DNA sequences of constructed vectors were confirmed by Sanger sequencing. To achieve genome-edited mcherry knock-in *emb-9(xyz9[emb-9::mcherry])*, we used PCR fragment for repair template. pJK750 (type IV collagen::mCherry expression vector) (Ihara *et al*, 2011) was used as PCR template using following primers: 5’ arm *emb-9* fwd TAAGTTTGCAGTCGTTAGTAAAG, 3’ arm *emb-9* rev AAGTTTGCAGTCGTTAGTAAAG. pDD162 (Peft-3::Cas9 + Empty sgRNA) vector from Bob Goldstein (Addgene plasmid # 47549) was used for Cas9 expression (Dickinson *et al*, 2013). The sgRNA plasmid was derived from Addgene plasmid 46169. For direct cleavage of target sequence, the following sgRNA sequences were used: 5’ CACAATGGAATTG/TCGGTCT 3’ for *nid-1*; and 5’AGAATTGTCAATCAA/GTTGC 3’ for *emb-9*. To prevent re-digestion after knock-in, fluorescence-tag was inserted into middle (/) position at sgRNA sequence at *nid-1* and *emb-9*, respectively. The sgRNA vectors were microinjected together with 50 ng/µL pDD162, 50 ng/µL sur-5::gfp, and 50 ng/µL repair templates in N2 animals. Single *mkate2::NID-1* was obtained based on roller phenotype, the SEC was excised as described (Dickinson & Goldstein, 2016). We selected candidate knock-in *emb-9::mcherry* strain, based on expression of EMB-9::mCherry, and confirmed the sequence of insertion sites. All images were acquired using an Axiocam 506 mono mounted on a Zeiss AxioImage A2 microscope equipped with a 20× Plan Apochromat objective lens that was controlled by ZEN 2.3 pro (Zeiss). Images were optimized and superimposed using Photoshop CS5 Extended (Adobe Systems).

Strain: IHR-168 *xyz8[mkate2::NID-1]*V

IHR-169 *xyz9[emb-9::mcherry]*III

It will be sent to the CGC.
